# Supplementary material for: Pyodermitis during Nivolumab Treatment for Non-Small Cell Lung Cancer: A Case Report and Review of the Literature
Source: Int J Mol Sci. 2023 Feb 26;24(5):4580. doi: 10.3390/ijms24054580 (PMC10003408; doi:10.3390/ijms24054580)
Supplement: Supplementary file 1 [file ijms-24-04580-s001.zip › ijms-2218080-supplementary.pdf]

Table S1. Antibiotic sensitivity testing. EUCAST MIC (mg/L) breakpoint: minimal inhibitor concentration; R: resistance. S: Susceptible, standard exposure.

Drug	MIC (mg/L)	Interpretation
**Fusidic acid**	<= 0.25	S
**ceftaroline**	<= 0.25	S
**Ceftaroline (pneumonie)**	<= 0.25	S
**Clindamycin**	0.25	**R**
**Daptomycin**	0.5	S
**Doxycycline**	<=0.5	S
**Erythromycin**	>4	**R**
**Gentamycin**	>4	**R**
**Levofloxacin**	<= 1	S
**Linezolid**	2	S
**Moxifloxacin**	0.25	S
**Mupirocin**	<= 1	S
**Oxacillin screening**	<= 0.25	S
**Rifampicin**	<= 0.062	S
**Tedizolid**	<= 0.25	S
**Teicoplanin**	<= 0.25	S
**Tigecycline**	<= 0.125	S
**Trimethoprim/sulpham.**	1	S
**Vancomycin**	<= 0.5	S

Figure S1. High-resolution images a) Chest x-ray (Thoravision). Examination performed with digital technique in the two orthogonal projections. The parenchymal opacity already indicated on the right and better evident in the previous TC of 3/3/2015 is confirmed. Disventilatory streak in the left basal site. b) Examination performed before and after intravenous administration of organ-iodized contrast medium place comparison with the previous PET-CT examination carried out on 29/7/2016. Compared to the previous one, there is a documented increase in the size of the newly formed tissue located in right hilar seat (current dimensions 27 x 25 mm vs 20 x 11 mm of the previous exam). The findings in the lungs are unchanged, in particular the thickening area previously described at the level of the LID. Not pleural effusion flaps c) Examination carried out before and after administration of organ-iodized MDC by IV route, compared with previous PET-TC carried out on 5/7/2018. There are no areas of pathological enhancement in the sub and supratentorial sites. Axial ventricular system, of dimensions within the limits of the norm. The results of the surgery known in the anamnesis in the thoracic area on the right are substantially confirmed unchanged. The millimetric nodular formation already evident at the lower lobe appears unchanged right in the previous aforementioned (series 3-image 328). Signs of emphysema in both parenchymal areas. No pleural effusion flaps are observed. d) Neck-thorax: Nodular thyroid isthmic formation requiring monitoring remains unchanged instrumental with ultrasound examination. The results of surgery on the upper right lobe are confirmed. The share of tissue in the right hilar seat and the solid nodular formation (4 mm) in correspondence of the LID [series 301 imm 225]. No pleural or pericardial effusion. Pervie the main airways. Sub-centimetric lymph node formations in the hilar and mediastinal region were stable in size.

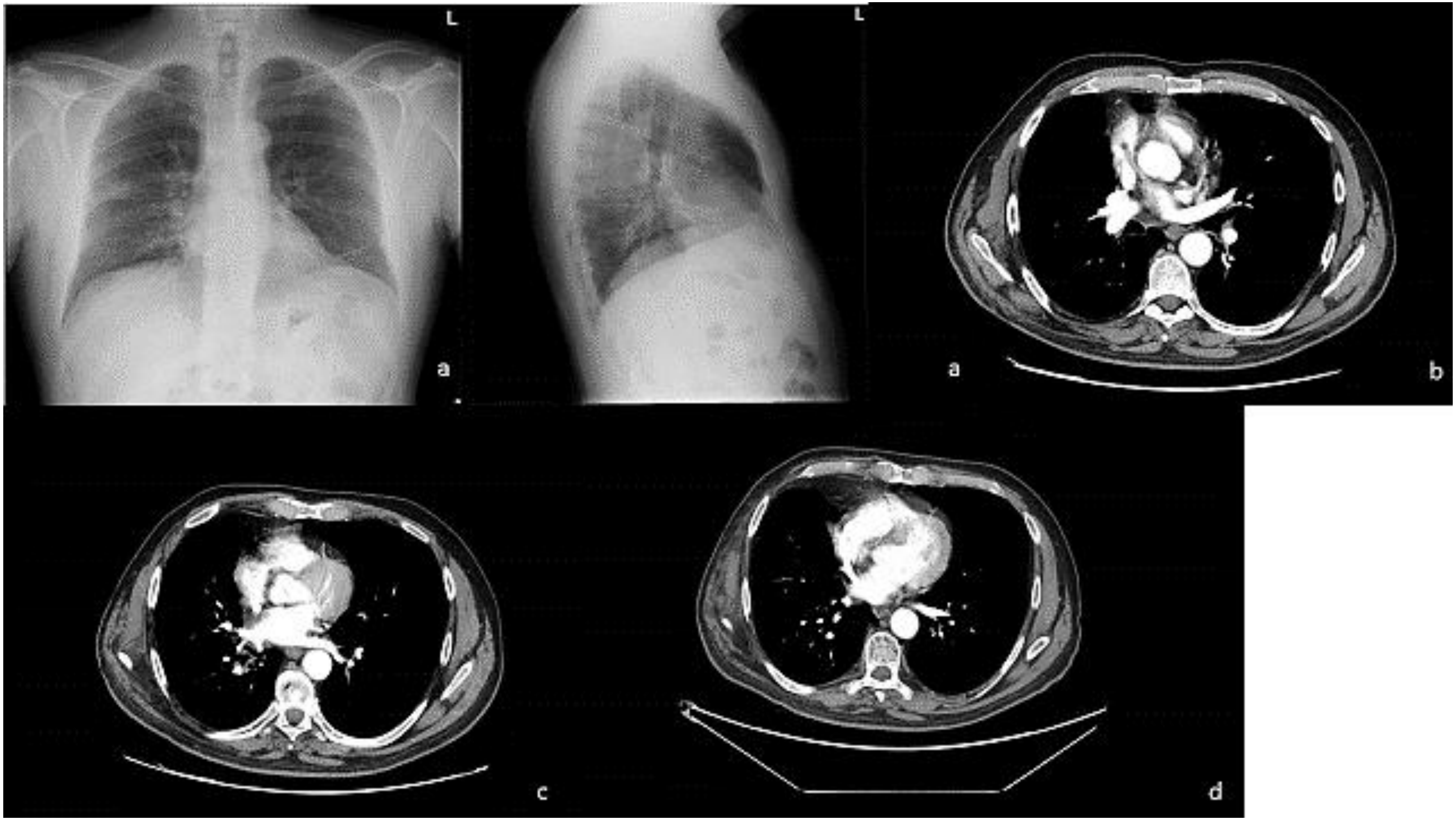
L
L
a
a
b
c
d

Search Strategy

We performed a comprehensive search in the following databases from 2015 to December 2022: Cochrane Central Register of Controlled Trials; MEDLINE; Embase; US National Institutes of Health Ongoing Trials Register; NIHR Clinical Research Network Portfolio Database; and the World Health Organization International Clinical Trials Registry Platform. We studied reference lists and published systematic review articles. We used the term "nivolumab" with the following keywords, in combination: "cutaneous infection", "*Staphylococcus spp.*". Only English language articles were included in the searches. Forward citation searching of the reference lists of the original studies and review articles was also conducted.

Inclusion Criteria

To investigate the relationship between nivolumab and staphylococcal cutaneous infections, if the study included nivolumab with other drugs, only the nivolumab frame was analyzed. All human studies were included, with no restrictions on age, sex, ethnicity or type of study. Case reports and case series were included if they described staphylococcal cutaneous infections during nivolumab treatment.

Exclusion Criteria

The target intervention excluded the analyses of other pathologies not due to fungal pathogens, and non-English language articles.

Result

Forty-two articles or trials regarding the relationship between nivolumab and staphylococcal cutaneous infections were identified by this quantitative research. Thirty-eight were excluded after the application of exclusion criteria. Among the four articles or trials eligible for evaluation, one was excluded after abstract or full text reading. Three articles or trials were evaluated in this case report (Figure S2). The results of our research are summarized in Table 1.
